# WSES classification and guidelines for liver trauma

**DOI:** 10.1186/s13017-016-0105-2

**Published:** 2016-10-10

**Authors:** Federico Coccolini, Fausto Catena, Ernest E. Moore, Rao Ivatury, Walter Biffl, Andrew Peitzman, Raul Coimbra, Sandro Rizoli, Yoram Kluger, Fikri M. Abu-Zidan, Marco Ceresoli, Giulia Montori, Massimo Sartelli, Dieter Weber, Gustavo Fraga, Noel Naidoo, Frederick A. Moore, Nicola Zanini, Luca Ansaloni

**Affiliations:** 1 General Emergency and Trauma Surgery Department, Papa Giovanni XXIII Hospital, Piazza OMS 1, 24127 Bergamo, Italy; 2 Emergency and Trauma Surgery, Parma Maggiore Hospital, Parma, Italy; 3 Trauma Surgery, Denver Health, Denver, CO USA; 4 Virginia Commonwealth University, Richmond, VA USA; 5 Acute Care Surgery, The Queen’s Medical Center, Honolulu, HI USA; 6 Department of Surgery, Trauma and Surgical Services, University of Pittsburgh School of Medicine, Pittsburgh, USA; 7 Department of Surgery, UC San Diego Health System, San Diego, USA; 8 Trauma & Acute Care Service, St Michael’s Hospital, Toronto, ON Canada; 9 Division of General Surgery Rambam Health Care Campus, Haifa, Israel; 10 Department of Surgery, College of Medicine and Health Sciences, UAE University, Al-Ain, United Arab Emirates; 11 Department of Surgery, Macerata Hospital, Macerata, Italy; 12 Department of General Surgery, Royal Perth Hospital, Perth, Australia; 13 Faculdade de Ciências Médicas (FCM)-Unicamp, Campinas, SP Brazil; 14 Department of Surgery, University of KwaZulu-Natal, Durban, South Africa; 15 Department of Surgery, University of Florida, Gainesville, FL USA; 16 General Surgery Department, Infermi Hospital, Rimini, Italy

**Keywords:** Liver trauma, Minor, Moderate, Severe, Classification, Guidelines, Surgery, Hemorrage, Operative management, Non-operative management

## Abstract

The severity of liver injuries has been universally classified according to the American Association for the Surgery of Trauma (AAST) grading scale. In determining the optimal treatment strategy, however, the haemodynamic status and associated injuries should be considered. Thus the management of liver trauma is ultimately based on the anatomy of the injury and the physiology of the patient. This paper presents the World Society of Emergency Surgery (WSES) classification of liver trauma and the management Guidelines.

## Background

The severity of liver injuries is universally classified according to the American Association for the Surgery of Trauma (AAST) grading scale (Table 1) [1]. The majority of patients admitted for liver injuries have grade I, II or III and are successfully treated with nonoperative management (NOM). In contrast, almost two-thirds of grade IV or V injuries require laparotomy (operative management, OM) [2]. However in many cases there is no correlation between AAST grade and patient physiologic status. Moreover the management of liver trauma has markedly changed through the last three decades with a significant improvement in outcomes, especially in blunt trauma, due to improvements in diagnostic and therapeutic tools [3–5]. In determining the optimal treatment strategy, the AAST classification should be supplemented by hemodynamic status and associated injuries. The anatomical description of liver lesions is fundamental in the management algorithm but not definitive. In fact, in clinical practice the decision whether patients need to be managed operatively or undergo NOM is based mainly on the clinical conditions and the associated injuries, and less on the AAST liver injury grade. Moreover, in some situations patients conditions lead to an emergent transfer to the operating room (OR) without the opportunity to define the grade of liver lesions before the surgical exploration; thus confirming the primary importance of the patient’s overall clinical condition. Utimately, the management of trauma requires an assessment of the anatomical injury and its physiologic effects.

**Table 1:** AAST Liver Trauma Classification

Grade	Injury type	Injury description
I	Haematoma	Subcapsular <10 % surface
	Laceration	Capsular tear <1 cm parenchymal depth
II	Haematoma	Subcapsular 10–50 % surface area; intraprenchymal, <10 cm diameter
	Laceration	1–3 cm parenchymal depth, <10 cm in length
III	Haematoma	Subcapsular >50 % surface area or expanding, ruptured subcapsular or parenchymal haematoma. Intraprenchymal haematoma >10 cm
	Laceration	>3 cm parenchymal depth
IV	Laceration	Parenchymal disruption 25–75 % of hepatic lobe
	Vascular	Juxtavenous hepatic injuries i.e. retrohepatic vena cava/centrl major hepatic veins
VI	Vascular	Hepatic avulsion

Advance one grade for multiple injuries up to grade III

AAST liver injury scale (1994 revision)

This paper aims to present the World Society of Emergency Surgery (WSES) classification of liver trauma and the treatment Guidelines, following the WSES position paper emerged from the Second WSES World Congress [6].

As stated in the position paper, WSES includes surgeons from around the globe. This Classification and Guidelines statement aims to direct the management of liver trauma, acknowledging that there are acceptable alternative management options. In reality, not all trauma surgeons work in the same conditions and have the same facilities and technologies available [6].

## Methods

The discussion of the present guidelines started in 2011 during the WSES World Congress in Bergamo (Italy). From that first discussion, through the Delphi process came the published position paper [6]. A group of experts in the field coordinated by a central coordinator was contacted to express their evidence-based opinion on several issues about the liver trauma management differentiated into blunt and penetrating trauma and evaluating the conservative and operative management for both.

The central coordinator assembled the different answers derived from the first round and drafted the first version that was subsequently revised by each member of the expert group separately in the second round. The definitive version about which the agreement was reached consisted in the position paper published in 2013 [6].

In July 2013 the position paper was discussed during the WSES World Congress in Jerusalem (Israel) and then a subsequent round of consultation among a group of experts evaluated the associated WSES classification and the new evidence based improvements. Once reached the agreement between the first experts group, another round among a larger experts group lead to the present form of the WSES classification and guidelines of liver trauma to which all the experts agreed. Levels of evidence have been evaluated in agreement with the Oxford guidelines.

### WSES classification

The WSES position paper suggested dividing hepatic traumatic lesions into minor (grade I, II), moderate (grade III) and major/severe (grade IV, V, VI) [6]. This classification has not previously been clearly defined by the literature. Frequently low-grade AAST lesions (i.e. grade I-III) are considered as minor or moderate and treated with NOM [7, 8]. However some patients with high-grade lesions (i.e. grade IV-V laceration with parenchymal disruption involving more than 75 % of the hepatic lobe or more than 3 Couinaud segments within a single lobe) may be hemodynamically stable and successfully treated nonoperatively [2]. On the other hand, “minor” lesions associated with hemodynamic instability often must be treated with OM. This demonstrates that the classification of liver injuries into minor and major must consider not only the anatomic AAST classification but more importantly, the hemodynamic status and the associated injuries.

The Advanced Trauma Life Support (ATLS) definition considers as “unstable” the patient with: blood pressure <90 mmHg and heart rate >120 bpm, with evidence of skin vasoconstriction (cool, clammy, decreased capillary refill), altered level of consciousness and/or shortness of breath [9].

The WSES Classification divides Hepatic Injuries into three classes:

- Minor (WSES grade I).
- Moderate (WSES grade II).
- Severe (WSES grade III and IV).

The classification considers either the AAST classification either the hemodynamic status and the associated lesions (Table 2).

**Table 2:** WSES Liver Trauma Classification

	WSES grade	Blunt/Penetrating (Stab/Guns)	AAST	Haemodynamic	CT-scan	First-line Treatment
MINOR	WSES grade I	B/P SW/GSW	I-II	Stable		
MODERATE	WSES grade II	B/P SW/GSW	III	Stable	Yes + Local Exploration in SW#	NOM* + Serial Clinical/Laboratory/ Radiological Evaluation
SEVERE	WSES grade III	B/P SW/GSW	IV-V	Stable		
	WSES grade IV	B/P SW/GSW	I-VI	Unstable	No	OM

(*SW* Stab Wound, *GSW* Gun Shot Wound; OM: Operative Management; NOM: Non Operative Management; *NOM should only be attempted in centers capable of a precise diagnosis of the severity of liver injuries and capable of intensive management (close clinical observation and haemodynamic monitoring in a high dependency/intensive care environment, including serial clinical examination and laboratory assay, with immediate access to diagnostics, interventional radiology and surgery and immediately available access to blood and blood products; # wound exploration near the inferior costal margin should be avoided if not strictly necessary because of the high risk to damage the intercostal vessels)

*Minor hepatic injuries*:

- ***WSES grade I*** includes AAST grade I-II hemodynamically stable either blunt or penetrating lesions.

*Moderate hepatic injuries*:

- ***WSES grade II*** includes AAST grade III hemodynamically stable either blunt or penetrating lesions.

*Severe hepatic injuries*:

- ***WSES grade III*** includes AAST grade IV-VI hemodynamically stable either blunt or penetrating lesions.
- ***WSES grade IV*** includes AAST grade I-VI hemodynamically unstable either blunt or penetrating lesions.

**Fig. 1:**
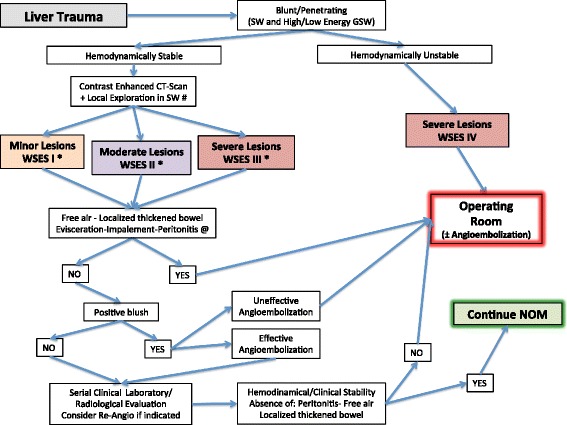
Liver Trauma Management Algorithm. (*SW* Stab Wound, *GSW* Gun Shot Wound; *NOM should only be attempted in centers capable of a precise diagnosis of the severity of liver injuries and capable of intensive management (close clinical observation and haemodynamic monitoring in a high dependency/intensive care environment, including serial clinical examination and laboratory assay, with immediate access to diagnostics, interventional radiology and surgery and immediately available access to blood and blood products; # wound exploration near the inferior costal margin should be avoided if not strictly necessary because of the high risk to damage the intercostal vessels; @ extremely selected patients hemodynamically stable with evisceration and/or impalement and/or diffuse peritonitis with the certainty of an exclusive and isolated abdominal lesion could be considered as candidate to be directly taken to the operating room without contrast enanched CT-scan)

Basing on the present classification WSES indicates a management algorithm explained in Fig. 1.

### Recommendations for non operative management (NOM) in blunt liver trauma (BLT)

*Blunt trauma patients with hemodynamic stability and absence of other internal injuries requiring surgery, should undergo an initial attempt of NOM irrespective of injury grade (GoR 2 A)*.

*NOM is contraindicated in the setting of hemodynamic instability or peritonitis (GoR 2 A)*.

*NOM of moderate or severe liver injuries should be considered only in an environment that provides capability for patient intensive monitoring, angiography, an immediately available OR and immediate access to blood and blood product (GoR 2 A)*.

*In patients being considered for NOM, CT-scan with intravenous contrast should be performed to define the anatomic liver injury and identify associated injuries (GoR 2 A)*.

*Angiography with embolization may be considered the first-line intervention in patients with hemodynamic stability and arterial blush on CT-scan (GoR 2 B)*.

In hemodynamically stable blunt trauma patients without other associated injuries requiring OM, NOM is considered the standard of care [10–12]. In case of hemodynamic instability or peritonitis NOM is contraindicated [7, 11, 13].

The requirements to attempt NOM of moderate and severe injuries are the capability to make a diagnosis of the severity of liver injuries, and to provide intensive management (continuous clinical monitoring, serial hemoglobin monitoring, and around-the-clock availability of CT-scan, angiography, OR, and blood and blood products) [14–19]. No evidence exists at present to define the optimal monitoring type and duration.

In patients with ongoing resuscitative needs, the angioembolization is considered as an “extension” of resuscitation. However with the aim to reduce the need for transfusions and surgery, angioembolization can be applied safely but generally only in selected centers [13, 20, 21]. If required it can be safely repeated. Positive results associated with its early use have been published [22, 23].

In blunt hepatic trauma, particularly after high-grade injury, complications occur in 12–14 % of patients [13, 24]. Diagnostic tools for complications after NOM include: clinical examination, blood tests, ultrasound and CT-scan. Although routine follow-up with CT-scan is not necessary, [2, 13, 24] in the presence of abnormal inflammatory response, abdominal pain, fever, jaundice or drop of hemoglobin level, CT-scan is recommended [13]. Bleeding, abdominal compartment syndrome, infections (abscesses and other infections), biliary complications (bile leak, hemobilia, bilioma, biliary peritonitis, biliary fistula) and liver necrosis are the most frequent complications associated with NOM [14, 24]. Ultrasound is useful in the assessment of bile leak/biloma in grade IV-V injuries, especially with a central laceration.

Re-bleeding or secondary hemorrhage are frequent (as in the rupture of a subcapsular hematoma or a pseudo-aneurysm) [13, 24]. In the majority of cases (69 %), “late” bleeding can be treated non-operatively [13, 24]. Post-traumatic hepatic artery pseudo-aneurysms are rare and they can usually be managed with selective embolization [6, 25].

Biliary complications can occur in 30 % of cases. Endoscopic retrograde cholangio-pancreatography (ERCP) and eventual stenting, percutaneous drainage and surgical intervention (open or laparoscopic) are all effective ways to manage biliary complications [13]. In presence of intrahepatic bilio-venous fistula (frequent associated with bilemia) ERCP represents an effective tool [26].

CT-scan or ultrasound-guided drainage are both effective in managing peri-hepatic abscesses (incidence 0–7 %) [13, 22, 24]. In presence of necrosis and devascularization of hepatic segments surgical management would be indicated [6, 24]. Hemobilia is uncommon and frequently associated with pseudo-aneurysm [2, 6, 24]. In hemodynamically stable and non-septic patients embolization is safe and could be considered as the first approach; otherwise surgical management is mandatory [6, 24].

Lastly, the liver compartment syndrome is rare and has been described in some case reports as a consequence of large sub-capsular hematomas. Decompression by percutaneous drainage or by laparoscopy has been described [24, 27].

No standard follow-up and monitoring protocol exist to evaluate patients with NOM liver injuries [6]. Serial clinical evaluation and hemoglobin measurement are considered the pillars in evaluating patients undergone to NOM [10]. Abdominal ultrasound could help in managing non-operatively managed liver trauma patients.

### Recommendations for NOM in penetrating liver trauma (PLT)

*NOM in penetrating liver trauma could be considered only in case of hemodynamic stability and absence of: peritonitis, significant free air, localized thickened bowel wall, evisceration, impalement (GoR 2 A)*.

*NOM in penetrating liver trauma should be considered only in an environment that provides capability for patient intensive monitoring, angiography, an immediately available OR and immediate access to blood and blood product (GoR 2 A)*.

*CT-scan with intravenous contrast should be always performed to identify penetrating liver injuries suitable for NOM (GoR 2 A)*.

*Serial clinical evaluations (physical exams and laboratory testing) must be performed to detect a change in clinical status during NOM (GoR 2 A)*.

*Angioembolisation is to be considered in case of arterial bleeding in a hemodynamic stable patient without other indication for OM (GoR 2 A)*.

*Severe head and spinal cord injuries should be considered as relative indications for OM, given the inability to reliably evaluate the clinical status (GoR 2A)*.

The most recent published trials demonstrate a high success rate for NOM in 50 % of stab wounds (SW) in the anterior abdomen and in about 85 % in the posterior abdomen [6, 28]. The same concept has also been applied to gunshot wounds (GSWs) [29, 30]. However, a distinction should be made between low and high-energy penetrating trauma in deciding either for OM or NOM. In case of low energy, both SW and GSW, NOM can be safely applied. High energy GSW and other ballistic injuries are less amenable to NOM because of the high-energy transfer, and in 90 % of cases an OM is required [6, 31, 32]. Of note, a 25 % non-therapeutic laparotomy rate is reported in abdominal GSWs [31]. This confirms that in selective cases NOM could be pursued either in GSWs.

Clinical trials report a high success rate of NOM in penetrating liver injuries (69 to 100 %) [29, 30, 32–37]. Absolute requirements for NOM are: hemodynamic stability, absence of peritonitis, and an evaluable abdomen [6]. Evisceration and impalement are other indications for OM [30, 32, 34]. Current guidelines suggest that hemodynamically stable patients presenting with evisceration and/or impalement and/or diffuse peritonitis should be considered candidates to be directly taken to the OR without CT-scan [30]. These findings are particularly important in cases of gunshot injuries. Other suggested predictive criteria of NOM failure in abdominal GSWs according to Navsaria et al. are: associated head and spinal cord injuries (that preclude regular clinical examination) and significant reduction in hemoglobin requiring more than 2–4 units of blood transfusion in 24 h [6, 29].

In SWs the role of CT scan has been questioned [28, 34]. Local wound exploration (LWE) is considered accurate in determining the depth of penetration; sometimes in little wounds it would be necessary to enlarge a little the incision [6, 30]. However, wound exploration near the inferior costal margin should be avoided if not strictly necessary because of the high risk to damage the intercostal vessels. Emergency laparotomy has been reported to be necessary even in some cases with negative CT-scan [34]. CT-scan may be necessary in obese patients and when the wound tract is long, tangential and difficult to determine the trajectory [6, 34].

In NOM of GSWs the CT-scan can help in determining the trajectory. However not all authors consider it mandatory [29, 31]. Velmahos et al. reported a CT-scan specificity of 96 % and a sensitivity of 90.5 % for GSWs requiring laparotomy [38]. The gold standard to decide for OM or NOM remains the serial clinical examination [6, 31].

NOM is contraindicated in case of CT-scan detection of free intra- or retro-peritoneal air, free intra-peritoneal fluid in the absence of solid organ injury, localized bowel wall thickening, bullet tract close to hollow viscus with surrounding hematoma [33] and in high energy penetrating trauma. In NOM strict clinical and hemoglobin evaluation should be done (every 4–6 h for at least 48 h); once stabilized the patient could be transferred to the ward [28, 29, 34].

There is considerable variation in local CT-scan imaging practices, and no uniform standard exists. Variations are dependent on imaging hardware, radiation exposure, contrast dose, and image sequences, among other factors. For example, image acquisition may occur in a triphasic fashion (non-contrast, arterial, and portal venous phases), or as a single phase following a split bolus contrast injection, providing a mixed arterial and portal venous phase. These variables have not been standardized across centers, or in the literature, and require expert radiologist consideration and manipulation for optimal diagnostic yield, and are dependent on the study indication.

Even in penetrating liver trauma, the angioembolization is considered as an “extension” of resuscitation in those patients presenting with ongoing resuscitative needs. However angioembolization can be applied safely only in selected centers [13, 20, 21]. If required it can be safely repeated.

The main reluctance of surgeons to employ NOM in penetrating trauma is related to the fear of missing other abdominal lesions, especially hollow viscus perforation [6, 33]. Published data clearly showed that in patients without peritonitis on admission, no increase in mortality rates with missed hollow viscus perforation has been reported [39]. On the other hand, non-therapeutic laparotomy has been demonstrated to increase the complication rate [39]. Nevertheless OM in penetrating liver injuries has a higher liver-related complication rate (50–52 %) than in blunt ones [6, 33].

### Concomitant severe head injuries

The otimal management of concomitant severe head and liver injuries is debated. In patients with severe head injuries hypotension may be deleterious, and OM could be suggested as safer [24, 36]. Recently, a large cohort of 1106 non-operatively managed low-energy gunshot liver injuries, has been published by Navsaria et al. [36]. The presence of concomitant liver and severe head injuries has been considered one of the main exclusion criteria to NOM. Authors stated that: “Hemodynamically stable patients with unreliable clinical examinations (head and/or high spinal cord injury) must also undergo an urgent exploratory laparotomy”. Another paper analyzing 63 patients by Navsaria et al. suggested as predictive criteria for NOM failure in abdominal low-energy GSWs is the association with head and spinal cord injuries precluding meaningful clinical examination [29].

### Follow-up after successful NOM

Clear and definitive direction for post-injury follow-up and normal activity resumption in those patients who experienced NOM haven’t been published yet. General recommendations are to resume usual activity after 3–4 months in patients with an uncomplicated hospital course. This derives from the observation that the majority of liver lesions heal in almost 4 months [10, 24]. If the CT-scan follow-up (in grade III-V lesions) has shown significant healing normal activity can be resumed even after 1 month [24].

Patients should to be counseled not to remain alone for long periods and to return to the hospital immediately if they experience increasing abdominal pain, lightheadedness, nausea or vomiting [6, 10].

### Recommendations for operative management (OM) in liver trauma (blunt and penetrating)

*Patients should undergo OM in liver trauma (blunt and penetrating) in case of hemodynamic instability, concomitant internal organs injury requiring surgery, evisceration, impalement (GoR 2 A).*

*Primary surgical intention should be to control the hemorrhage, to control bile leak and to institute an intensive resuscitation as soon as possible (GoR 2 B).*

*Major hepatic resections should be avoided at first, and considered subsequently (delayed fashion) only in case of large devitalized liver portions and in centers with the necessary expertise (GoR 3 B)*.

*Angioembolisation is a useful tool in case of persistent arterial bleeding (GoR 2 A)*.

As exsanguination represents the leading cause of death in liver injuries OM decision mainly depends on hemodynamic status and associated injuries [6].

In those cases where no major bleeding are present at the laparotomy, the bleeding may be controlled by compression alone or with electrocautery, bipolar devices, argon beam coagulation, topical hemostatic agents, or omental packing [6, 8, 24, 40, 41].

In presence of major haemorrhage more aggressive procedures can be necessary. These include first of all hepatic manual compression and hepatic packing, ligation of vessels in the wound, hepatic debridement, balloon tamponade, shunting procedures, or hepatic vascular isolation. It is important to provide concomitant intraoperative intensive resuscitation aiming to reverse the lethal triad [6, 8, 41].

Temporary abdominal closure can be safely considered in all those patients when the risk of developing abdominal compartment syndrome is high and when a second look after patient’s hemodynamic stabilization is needed [8, 40, 41].

Anatomic hepatic resection can be considered as a surgical option [2, 42, 43]. In unstable patients and during damage control surgery a non-anatomic resection is safer and easier [6, 8, 24, 44]. For staged liver resection, either anatomic either non-anatomic ones can be safely made with stapling device in experienced hands [44].

If despite the fundamental initial maneuvers (hepatic packing, Pringle maneuver) the bleeding persists and evident lesion to a hepatic artery is found, an attempt to control it should be made. If repair is not possible a selective hepatic artery ligation can be considered as a viable option. In case of right or common hepatic artery ligation, cholecystectomy should be performed to avoid gallbladder necrosis [44, 45]. Post-operative angio-embolization is a viable option, when possible, allowing hemorrhage control while reducing the complications [6, 8, 24, 46]. After artery ligation, in fact, the risk of hepatic necrosis, biloma and abscesses increases [6].

Portal vein injuries should be repaired primarily. The portal vein ligation should be avoided because liver necrosis or massive bowel edema may occur. Liver Packing and a second look or liver resection are preferable to portal ligation [6, 44].

In those cases where Pringle maneuver or arterial control fails, and the bleeding persists from behind the liver, a retro-hepatic caval or hepatic vein injury could be present [6, 46]. Three therapeutic options exist: 1) tamponade with hepatic packing, 2) direct repair (with or without vascular isolation), and 3) lobar resection [7]. Liver packing is the most successful method of managing severe venous injuries [6, 24, 47–49]. Direct venous repair is problematic in non-experienced hands, with a high mortality rate [6, 24].

When hepatic vascular exclusion is necessary, different types of shunting procedures have been described, most of them anecdotally. The veno-veno bypass (femoral vein to axillary or jugular vein by pass) or the use of fenestrated stent grafts are the most frequent type of shunt used by surgeons familiar with their use [8, 24, 44, 50]. The atrio-caval shunt bypasses the retro-hepatic cava blood through the right atrium using a chest tube put into the inferior cava vein. Mortality rates in such a complicated situations are high [8]. Liver exclusion is generally poorly tolerated in the unstable patient with major blood loss [6].

In the emergency, in cases of liver avulsion or total crush injury, when a total hepatic resection must be done, hepatic transplantation has been described [44].

The exact role of post-operative angio-embolization is still not well defined [51–55]. Two principal indications have been proposed: 1) after primary operative hemostasis in stable or stabilized patients, with an evidence at contrast enhanced CT-scan of active bleeding, and 2) as adjunctive hemostatic control in patients with uncontrolled suspected arterial bleeding despite emergency laparotomy [6, 56].

## Conclusions

The management of trauma poses in definitive the attention in treating also the physiology and decision can be more effective when both anatomy of injury and its physiological effects are combined.

## Abbreviations

AAST: American Association for Surgery for Trauma
ATLS: Advanced Trauma Life Support
BLT: Blunt liver trauma
DCS: Damage Control Surgery
ERCP: Endoscopic retrograde cholangio-pancreatography
GSW: Gunshot wound
NOM: Non-Operative Management
OM: Operative Management
OR: Operating Room
SW: Stab wounds
WSES: World Society of Emergency Surgery
